# Dietary Patterns Associated with Sebum Content, Skin Hydration and pH, and Their Sex-Dependent Differences in Healthy Korean Adults

**DOI:** 10.3390/nu11030619

**Published:** 2019-03-14

**Authors:** Sunhee Lim, Jihye Shin, Yunhi Cho, Kun-Pyo Kim

**Affiliations:** Department of Medical Nutrition, Graduate School of East-West Medical Science, Kyung Hee University, Yongin-si 17104, Korea; by_catherine@naver.com (S.L.); hleany@nate.com (J.S.)

**Keywords:** dietary pattern, sebum content, skin hydration, skin pH, sex difference

## Abstract

Sebum content, skin hydration and acidic skin pH are major factors in maintaining skin health. Various nutrients are reported to influence skin health, but the effect of dietary patterns (DPs) on skin health is unclear. In this study, we considered the DPs associated with these three skin health parameters in 84 healthy adults aged 19–37 years. Dietary intake was assessed using a food frequency questionnaire (FFQ) and skin health parameters were determined on the forehead of each subject. Among the four DPs extracted from the FFQ, DP2, characterized by a high intake of cereals, potatoes and starch, saccharides and fish and shellfish, was negatively associated with skin hydration. DP3, characterized by a high intake of potatoes and starch, seeds and nuts, fruits and eggs, was positively associated with acidic skin pH only before adjusting for potential confounders. On the other hand, DP4, characterized by a low intake of beans, and a high intake of meats, dairy products and beverages and alcohol, was negatively associated with acidic skin pH and positively associated with sebum content. The data stratified by sex revealed a negative association between skin hydration and DP2 in males and a negative association between sebum content and DP3 and a positive association between sebum content and DP4 in females. In conclusion, we demonstrated that specific DPs were associated with sebum content, skin hydration and pH in healthy Korean adults and that those associations were affected by sex.

## 1. Introduction

Human skin is primarily responsible for protecting the body from a loss of water and various threats from the external environment. Therefore, it is important to appropriately maintain the skin permeability barrier function, which can be affected by several parameters, including sebum content, skin hydration and skin pH [1]. Sebum, which provides antioxidants and antimicrobial lipids to the skin surface, enhances the skin permeability barrier function, but excess sebum can cause acne vulgaris [2]. A robust skin permeability barrier is indispensable to preserving skin hydration, which is crucial in the normal functioning and physiology of skin [3]. In addition, acidic skin pH is an important factor in maintaining the integrity of the skin permeability barrier [4]. It has been reported that skin pH is increased in skin diseases such as atopic dermatitis [4,5]. Thus, these three skin health parameters (sebum content, skin hydration and skin pH) and the skin permeability barrier function interact with one another, and those interactions are an important factor in maintaining skin homeostasis and health.

The function of the skin permeability barrier is also influenced by various internal and external factors, one of which is nutrition. It is well known that keratinocyte proliferation and differentiation in the epidermis is regulated by various nutrients, such as vitamin C [6,7], calcium [8] and linoleic acid (LA; 18:2 n-6) [9]. In addition, increasing evidence suggests that supplementation with those nutrients or functional foods such as borage oil [10] and probiotics [11] can improve skin health, including skin hydration and pH. Recently, several studies offered new insights into the association between regularly ingested foods and skin health. Specifically, high sebum content and the aggravation of acne vulgaris could be strongly influenced by food intake, including high glycemic-load diets and milk products, which induce the release of insulin and insulin-like growth factor (IGF)-1 [12,13,14]. However, the relationship between diets and skin hydration and pH remains unclear due to a lack of research. Furthermore, most studies on the relationship between diet and skin health parameters, including sebum content, skin hydration, and skin pH, are limited to the effect of a single food or nutrient rather than combined foods and nutrients. As people ingest food in complex combinations, dietary patterns could provide practical insights into the effects of diet on skin health [15]. However, to our knowledge, no study has yet examined the relationship between dietary patterns and skin health parameters. Therefore, in this study we investigated the dietary patterns associated with skin health parameters (sebum content, skin hydration, and skin pH) in healthy Korean adults.

## 2. Materials and Methods

### 2.1. Study Design

This cross-sectional observation study was approved by the Institutional Review Board of Kyung-Hee University (KHSIRB-18-016) and performed in accordance with the principles of the Declaration of Helsinki. Eighty-four healthy male (*n* = 44) and female (*n* = 40) subjects aged 19 to 37 years participated in this study and provided their informed consent after receiving an explanation of the study. The exclusion criteria in the subject recruitment were having a chronic disease or skin disorder such as psoriasis or atopic dermatitis and taking nutritional supplements or medication for the skin, such as retinoids or steroids. All questionnaires and measurements were completed in December 2016. The general characteristics and dietary data for the subjects were collected by questionnaire. Anthropometric data and skin health parameters were measured using non-invasive methods, as described below.

### 2.2. General Characteristics and Anthropometric Variables

Demographic and general health characteristics of the subjects (sex, age, smoking, drinking and physical activity) were collected by questionnaire. Smoking and drinking behavior was divided into two categories based on current status: current smokers/drinkers and current non-smokers/drinkers. Physical activity was also classified into two categories: regular exercisers (those who reported regularly exercising at least once per week) and non-exercisers. Height (cm) and weight (kg) were measured using standard methods with the participants wearing light clothing and no shoes. Body mass index (BMI) was calculated as weight (kg) divided by the square of height (m^2^).

### 2.3. Dietary Intake Assessment

Dietary intake was assessed using a simple semi-quantitative food frequency questionnaire (FFQ) that listed 63 food items regularly ingested by Koreans [16]. The subjects were asked to complete the questionnaire regarding their food intake frequency (with a standard portion size) during the past 12 months, and this process was overseen by trained interviewers. The frequency of food intake was categorized using a 9-point scale (3 times per day, 2 times per day, once per day, 3–4 times per week, 1–2 times per week, 2–3 times per month, once per month, 6 times per year, seldom or never). The FFQ results were converted to indicate the daily intake of food items or nutrients using the Computer Aided Nutritional Analysis Program (CAN-Pro) 5.0 developed by the Korean Nutrition Society. The daily intake of 16 food groups was also calculated with this software and used to develop the dietary patterns.

### 2.4. Skin Health Parameter Measurement

The subjects were not allowed to use any cosmetics on the measurement region on the test day. The measurements of skin health parameters were made after the subjects had remained in a resting position for 30 min in a room with a temperature of 22 ± 2 °C and a humidity of 40%–60%. All skin health parameters were assessed using non-invasive and well-established measuring methods (Courage & Khazaka, Cologne, Germany) [17,18,19]. Sebum content was determined using a sebumeter (SM815, Courage & Khazaka) based on the principle of grease spot photometry. The disposal tape of a sebumeter was placed against the forehead for 30 s to collect sebum. After 30 s, the transparency of the tape absorbing the sebum was measured photometrically and calculated as μg sebum/cm^2^. Skin hydration and skin pH were also determined on the forehead using a corneometer (CM820, Courage & Khazaka), which measures the electrical capacity of the skin surface, and a skin-pH-meter (PH900, Courage & Khazaka) equipped with a planar glass electrode and based on potentiometric determination, respectively. The skin hydration results were given in arbitrary units. All measurements were repeated in triplicate.

### 2.5. Statistical Analyses

Factor analysis (principle component) with varimax rotation was used to extract dietary patterns from the 16 food groups. As a minimum of five subjects per variable is recommended for conducting a factor analysis [20], we used food groups instead of food items. To determine the number of factors, we considered the natural interpretability of the factors using the eigenvalue (>1.0) and a scree plot test. Food groups with factor-loading absolute values >0.4 were considered to be the major contributors to each pattern. The factor scores for each derived pattern were calculated by summing the products between the intake of the food groups and their factor loadings, and they were categorized by quartiles (Q1–Q4) to examine the association between the dietary patterns and skin health parameters.

We performed a one-way analysis of variance for continuous variables and the chi-square test for categorical variables to compare the general characteristics across the quartiles of dietary pattern scores. A general linear model was used to determine linear trends in skin health parameter values across the quartiles of dietary pattern scores after adjusting for potential confounders. The first model was adjusted only for energy intake, and the second model was further adjusted for sex, age, BMI, smoking and physical activity. The adjusted means for skin health parameters and the 95% confidential intervals were also calculated. To check for sex effects on the relationship between skin health parameters and dietary pattern scores, we stratified the subjects into male and female and then analyzed the general linear model using data separated by sex after adjusting for age, BMI, smoking and physical activity. To understand the nutritional characteristics of the dietary patterns, we analyzed partial correlations of dietary pattern scores with the intake of the major nutrients, specific vitamins [21,22,23], omega-6 fatty acids [24,25,26] and amino acids [14,27] known to be related to skin health parameters.

IBM SPSS version 23.0 (IBM Corp, Armonk, NY, USA) and R statistical software version 3.4.3 (http://www.r-project.org/) were used for the statistical analyses in this study. Statistical significance was set at *p* = 0.05. 

## 3. Results

### 3.1. Dietary Patterns

Based on our factor analysis with 16 food groups, we extracted four major dietary patterns that accounted for 61.1% of the total variance in food groups (Table 1). The first dietary pattern (DP1), which accounted for 24.7% of the variance, was characterized by a high intake of beans, vegetables, mushrooms, meats, seaweeds, fat and oils and condiments (absolute factor loading >0.4). The second dietary pattern (DP2), which accounted for 14.0% of the variance, was characterized by a high intake of cereals, potatoes and starch, saccharides and fish and shellfish. The third dietary pattern (DP3), which accounted for 13.6% of the variance, was characterized by a high intake of potatoes and starch, seeds and nuts, fruits and eggs. The fourth dietary pattern (DP4), which accounted for 8.7% of the variance, was characterized by a low intake of beans and a high intake of meats, dairy products and beverages and alcohol.

### 3.2. General Characteristics by the Quartiles of Dietary Pattern Scores

The overall study population contained 44 males (52.4%) and 40 females (47.6%), and the average age was 24.8 ± 4.1 years (means ± SD). The average BMI was 23.2 ± 2.6 kg/m^2^, which is in the normal range for Asian populations (18.5 < BMI < 25) [28]. The average results from the skin health parameters in the overall study population were 144.9 ± 38.6 μg/cm^2^ for sebum content (normal range: 100–220) [29], 64.1 ± 7.6 for skin hydration (normal range: >40) [30], and 5.23 ± 0.43 for skin pH (normal range: 4.0–5.5) [31]. All values were within the normal range. The general characteristics based on the quartiles of dietary pattern scores are displayed in Table 2. Subjects with high scores (Q4) for DP1 or DP2 were more commonly male than female and more physically active with a higher energy intake than the subjects with low scores (Q1) for DP1 or DP2. In addition, subjects with high scores for DP1 were older than the subjects with low scores for DP1. Subjects with high scores for DP3 were older, drank less alcohol and exercised more than the subjects with low scores for DP3, and subjects with high scores for DP4 were more likely to be male and drank more alcohol than the subjects with low scores for DP4. There were no significant differences in the average BMI or the proportion of current smokers across the quartiles in any dietary pattern.

### 3.3. Association of Skin Health Parameters with Dietary Patterns

To evaluate the association of skin health parameters with the four dietary patterns, we analyzed alterations in sebum content, skin hydration and skin pH across the quartiles of dietary pattern scores after adjusting for potential confounders (Table 3). Model 1 was adjusted only for energy, and Model 2 was additionally adjusted for sex, age, BMI, smoking and physical activity. DP1 showed no association with skin health parameters. On the other hand, subjects with high scores for DP2 tended to have a low skin hydration in both Model 1 (*p* for trend = 0.017) and Model 2 (*p* for trend = 0.028). High scores for DP3 tended to have a low skin pH (a positive association with acidic skin pH) only in Model 1 (*p* for trend = 0.045), whereas high scores for DP4 tended to have a high skin pH (a negative association with acidic skin pH) in both Model 1 (*p* for trend = 0.045) and Model 2 (*p* for trend = 0.039). Furthermore, high scores for DP4 tended to have a high sebum content in both Model 1 (*p* for trend = 0.008) and Model 2 (*p* for trend = 0.018).

As there were differences in the proportion of males and females across the quartiles in most dietary patterns (Table 2), we checked the sex effects in the association between skin health parameters and dietary patterns after adjusting for energy intake, age, BMI, smoking and physical activity (Table 4). When the subjects were stratified as male and female, the negative association between DP2 and skin hydration remained statistically significant only in males (*p* for trend = 0.003), and the positive association between DP4 and sebum content remained significant only in females (*p* for trend = 0.026). The associations between DP3 and DP4 and skin pH did not remain statistically significant in either males or females. Furthermore, we additionally found that females with high scores for DP3 tended to have a low sebum content (*p* for trend = 0.009). These results indicate that the effects of diet on skin health parameters might differ between males and females.

### 3.4. Nutrient Intake by Dietary Patterns

To understand the nutritional characteristics of the dietary patterns that showed statistically significant associations with the skin health parameters (DP2 with skin hydration, DP3 with sebum and skin pH, and DP4 with sebum), we analyzed the partial correlations of those scores with the intake of nutrients known to be related to skin health parameters (major nutrients, specific vitamins [21,22,23], omega-6 fatty acids [24,25,26] and amino acids [14,27]) after adjusting for energy intake (Table 5). DP2 showed a positive association with carbohydrate intake (r = 0.475, *p* < 0.001) and a negative association with most of the other nutrients: protein (r = −0.406, *p* < 0.001), fat (r = −0.424, *p* < 0.001), fiber (r = −0.220, *p* = 0.046), vitamin A (r = −0.454, *p* < 0.001), vitamin C (r = −0.225, *p* = 0.041), vitamin D (r = −0.300, *p* = 0.006), LA (r = −0.302, *p* = 0.005), γ-linolenic acid (GLA, 18:3 n-6) (r = −0.395, *p* < 0.001), isoleucine (r = −0.221, *p* = 0.044), leucine (r = −0.247, *p* = 0.024) and valine (r = −0.243, *p* = 0.027). On the other hand, DP3 showed a negative association with leucine intake (r = −0.225, *p* = 0.040) and a positive association with most other nutrients: fiber (r = 0.556, *p* < 0.001), vitamin A (r = 0.396, *p* < 0.001), vitamin C (r = 0.685, *p* < 0.001), vitamin E (r = 0.454, *p* < 0.001), vitamin D (r = 0.627, *p* < 0.001), LA (r = 0.716, *p* < 0.001), GLA (r = 0.732, *p* < 0.001) and dihomo-γ-linolenic acid (DGLA, 20:3 n-6) (r = 0.692, *p* < 0.001). DP4 showed a negative association with the intake of carbohydrates (r = −0.426, *p* < 0.001), fiber (r = −0.390, *p* < 0.001), LA (r = −0.254, *p* = 0.020), and GLA (r = −0.224, *p* = 0.042) and a positive association with the intake of vitamin C (r = 0.218, *p* = 0.047), vitamin D (r = 0.281, *p* = 0.010), isoleucine (r = 0.436, *p* < 0.001), leucine (r = 0.470, *p* < 0.001), tryptophan (r = 0.415, *p* < 0.001), and valine (r = 0.456, *p* < 0.001).

## 4. Discussion

In this study we extracted four dietary patterns from the FFQs of our subjects and found that three of them were associated with sebum content, skin hydration and skin pH (Table 3). Furthermore, we found that those associations differed between males and females (Table 4). When we stratified the data by sex, we revealed that skin hydration and sebum content were associated with diet in males and females, respectively, and that skin pH was not associated with diet in males or females. Though the precise mechanism of those sex differences is uncertain, one possible explanation is hormonal differences between males and females. It has been reported that sex hormones, representatively androgen and estrogen, affect sebaceous gland function and epidermal barrier homeostasis [32]. Therefore, it is possible that diet could differently affect skin physiology, especially skin hydration and sebum, in males and females because of differential hormonal susceptibilities and the bioavailability of nutrients.

Little previous research has considered a possible link between skin hydration and diet. In this study, we found an association between a specific dietary pattern and skin hydration. According to our results (Table 3), DP2, characterized by a high intake of cereals, potatoes and starch, saccharides, and fish and shellfish, showed a negative association with skin hydration overall and in males. A high intake of carbohydrates, especially sugars, was previously reported to affect skin aging through the glycation of skin proteins, which decreases skin cell viability and induces inflammation [33]; those processes could be related to the deterioration in skin hydration we observed. On the other hand, although long-chain omega-3 fatty acids in fish are generally considered to positively influence skin health through their anti-inflammatory effects [34], the effect of fish intake on skin hydration is unclear. Therefore, to identify it, we further analyzed the association between skin hydration and the intake of the individual food groups in DP2 (data not shown). After adjusting for potential confounders in that analysis, skin hydration was not associated with the intake quartiles of fish and shellfish (*p* for trend = 0.984), but it showed a decreasing tendency across the intake quartiles of saccharides (*p* for trend = 0.040), indicating that the negative association between DP2 and skin hydration might result from the high intake of saccharides, not fish and shellfish. In addition, although energy intake tended to increase across the quartiles of DP2 scores (Table 2), the intake of vitamins A, C, and D, LA and GLA had a negative association with DP2 (Table 5). As supplementation with antioxidants [21,35,36], including beta-carotene, and vitamin C, or omega-6-fatty acids [26,36], including LA and GLA, has been reported to improve skin hydration and reduce dryness, the lack of those nutrients might also have influenced skin hydration, along with the high intake of sugars.

On the other hand, many studies have established the effect of diet on sebum and acne vulgaris. In particular, the Western diet, characterized by a high intake of total energy, sugars, meats and dairy products, has been reported to increase sebum synthesis and aggravate acne vulgaris [12]. High-glycemic diets, which contain a lot of simple sugars, lead to elevated levels of insulin and IGF-1, which in turn stimulate sebaceous gland hyperplasia and sebaceous lipogenesis [13,37]. In addition, milk proteins contain high amounts of tryptophan and the branch-chain amino acids (leucine, isoleucine, and valine) that also stimulate IGF-1 and insulin secretion [14,27]. Those previous studies agree with our results for DP4, which is characterized by a low intake of beans and a high intake of meats, dairy products and beverages (especially soft drinks) and alcohol, similar to the Western diet. Therefore, the positive association of DP4 with sebum content overall and in females could be attributable to the high intake of soft drinks, meat, and milk along with the high intake of tryptophan, leucine, isoleucine and valine, and the low intake of fiber (Table 5), which could stimulate insulin/IGF-1 secretion. In addition, the low intake of beans in DP4 could play a part in the sebum increase we observed because soybean isoflavones, which have weak estrogen-like effects, have been reported to alleviate acne vulgaris by decreasing the androgen levels that stimulate sebum synthesis [38]. Finally, DP4 included a high intake of alcohol (Table 1 and Table 2), which could also affect sebum content because alcohol intake has been reported to aggravate acne vulgaris [39].

On the other hand, DP3 showed a negative association with sebum content, even in females (Table 4). DP3, characterized by a high intake of potatoes and starch, seeds and nuts, fruits and eggs, showed a positive association with the intake of vitamins A, C, E and D, LA, GLA and DGLA (Table 5). In this regard, vitamins A [22] and D [23] and LA [24] were known to play a major role in regulating sebaceous gland physiology. Furthermore, although GLA cannot be produced from LA in the skin due to a lack of the needed desaturation enzyme [40], it could rapidly elongate into DGLA, whose oxidized metabolites are known to modulate the inflammatory process and hyperproliferative skin conditions [41]. Therefore, dietary supplementation with GLA has been reported to effectively improve acne vulgaris and decrease sebum production [25,41]. Therefore, those nutrients seem to be related to the negative association between DP3 and sebum content.

Keeping skin in an acidic pH range (4–6) is an important factor in maintaining skin health and preventing skin disease [42]; many studies have revealed that higher (less acidic) skin pH values are associated with skin aging [43] and pathological skin conditions, including atopic dermatitis [4,5] and acne vulgaris [44]. Nevertheless, little evidence is available on the association between food and nutrient intake and skin pH. In this study, we found that DP3 and DP4 were associated with skin pH (Table 3). DP3 showed a positive association with acidic skin pH before adjustment for potential confounders. This result agrees with our preliminary study, which found that a dietary pattern characterized by a high intake of seeds and nuts, and fruits was positively associated with acidic skin pH in healthy Korean adults [45]. Therefore, the antioxidants, vitamins and polyunsaturated fatty acids in seeds and nuts, and fruits seem to be related to improved skin pH. In this regard, we reported that increased skin pH could be reversed by dietary supplementation with borage oil containing LA and GLA in an essential fatty acid deficient guinea pig model [46], suggesting that omega 6 fatty acids, especially LA and GLA in seeds and nuts and eggs might play an important role in improving skin pH. On the contrary, DP4, which is similar to the Western diet, had a negative association with acidic skin pH in overall subjects. As explained above, DP4 could increase insulin and IGF-1 levels, which could lead to an increase in skin pH by inducing abnormal skin conditions such as the hyperproliferation of skin cells [47].

To the best of our knowledge, this is the first study to discover associations between dietary patterns and skin health parameters. To summarize our findings: DP2 was associated with decreased skin hydration (overall and males). DP3 was associated with decreased sebum content (females) and skin pH (overall), while DP4 was associated with increased sebum content (overall and females) and skin pH (overall). However, because the association between DP3 and skin pH was not significant after adjustment for potential confounders, further studies are required to clarify its association. In addition, the interpretation of our findings requires some care. First, because we considered only a relatively small number of subjects, our results have limited generalizability. Second, the causal relationship between dietary patterns and skin health parameters cannot be inferred from our results due to the cross-sectional nature of this study. Third, interpretations about the effects of a specific individual food or nutrient on skin health parameters requires attention because dietary patterns represent combinations of various food intakes. Particularly for nutrients that occur in small amounts, such as GLA and DGLA, the meaning of the associations we found requires careful interpretation. Finally, we measured skin health parameters on the forehead because it secretes more sebum than other sites. However, the face is highly influenced by various life habits, such as the use of cosmetics and other topical products and cleansers.

## 5. Conclusions

This study revealed that specific dietary patterns are related to the skin health parameters of sebum content, skin hydration and skin pH on the foreheads of healthy Korean adults. In addition, we found that those associations could differ by sex. These findings could be useful basic data for promoting skin health and preventing skin disease. However, further prospective large-scale studies and randomized controlled trials are needed to support these findings and address the limitations of this study.

## Figures and Tables

**Table 1:** Factor loading matrix for the dietary patterns.

Food Groups	Dietary Pattern *			
	DP1	DP2	DP3	DP4
Cereals	0.24 †	**0.79**		
Potatoes and starch		**0.63**	**0.46**	
Saccharides		**0.70**		
Beans	**0.43**		0.33	**−0.40**
Seeds and nuts	0.39		**0.64**	
Vegetables	**0.85**			
Mushrooms	**0.71**		0.36	
Fruits		0.22	**0.74**	
Meats	**0.61**			**0.44**
Eggs			**0.70**	
Fish and shellfish		**0.67**		
Seaweeds	**0.66**	0.30		
Dairy products			0.38	**0.50**
Fat and oils	**0.84**		0.27	
Beverages and alcohol				**0.80**
Condiments	**0.86**			−0.21

DP, dietary pattern; * Dietary patterns were extracted by factor analysis. † Only factors loading >|0.2| are displayed. The bold text indicates a factor loading >|0.4|.

**Table 2:** General characteristics across the quartiles of dietary pattern scores †.

Parameters	DP1					DP2					DP3					DP4				
	Q1	Q2	Q3	Q4	*p* for Trend	Q1	Q2	Q3	Q4	*p* for Trend	Q1	Q2	Q3	Q4	*p* for Trend	Q1	Q2	Q3	Q4	*p* for Trend
Number (*n*)	21	21	21	21		21	21	21	21		21	21	21	21		21	21	21	21	
Sex (male)					0.033 *					0.032 *					0.766					0.006 **
*n*	7	10	14	13		7	12	9	16		12	11	12	9		5	10	13	16	
%	33.3	47.6	66.7	61.9		33.3	57.1	42.9	76.2		57.1	52.4	57.1	42.9		23.8	47.6	61.9	76.2	
Age (years)					0.023 *					0.485					0.021*					0.281
Mean	23.5	24.4	25.1	26.3		25.0	25.8	24.0	24.6		24.1	23.9	24.4	27.0		26.0	24.2	25.0	24.2	
SD	4.4	3.6	3.7	4.4		4.6	4.1	4.1	3.5		4.5	2.7	3.7	4.6		4.3	3.5	4.7	3.7	
BMI (kg/m^2^)					0.429					0.555					0.281					0.061
Mean	22.8	23.5	22.9	23.7		22.8	23.2	23.6	23.2		23.0	22.8	23.1	23.9		22.7	22.4	24.0	23.7	
SD	2.7	2.7	2.1	3.0		2.9	2.8	2.1	2.7		2.8	2.8	2.5	2.4		2.6	2.1	2.7	2.8	
Current smoker					0.364					0.630					0.483					0.056
n	1	4	3	5		5	2	3	3		2	5	2	4		2	1	3	7	
%	4.8	19.0	14.3	23.8		23.8	9.5	14.3	14.3		9.5	23.8	9.5	19.0		9.5	4.8	14.3	33.3	
Current drinker					0.886					0.196					0.006 **					0.001 **
n	19	18	17	19		16	19	19	19		20	20	19	14		12	21	20	20	
%	90.5	85.7	81.0	90.5		76.2	90.5	90.5	90.5		95.2	95.2	90.5	66.7		57.1	100	95.2	95.2	
Regular exerciser ‡					0.015 *					0.049 *					0.049 *					0.728
n	15	13	16	21		13	17	16	19		15	13	18	19		17	15	18	15	
%	71.4	61.9	76.2	100.0		61.9	81.0	76.2	90.5		71.4	61.9	85.7	90.5		81.0	71.4	85.7	71.4	
Energy intake (kcal)					<0.001 ***					<0.001 ***					0.206					0.111
Mean	1460	1924	2327	2743		1667	1708	2244	2836		1994	1972	2246	2243		1911	2042	2223	2280	
SD	455	975	622	526		580	453	597	963		742	746	698	1051		656	1173	692	629	

DP, dietary pattern; BMI, body mass index; * *p* < 0.05, ** *p* < 0.01, *** *p* < 0.001; † Analysis of variance for continuous variables (presented as mean values and standard deviations) and chi-square analysis for categorical variables (presented as numbers and percentages), with a linear trend test across the quartiles of dietary pattern scores (Q1–Q4). ‡ Subjects reported regularly exercising at least once per week.

**Table 3:** Multivariate adjusted average of skin health parameters according to quartiles of dietary pattern scores †.

	Q1 (*n* = 21)		Q2 (*n* = 21)		Q3 (*n* = 21)		Q4 (*n* = 21)		*p* for Trend
	Mean	95% CI	Mean	95% CI	Mean	95% CI	Mean	95% CI	
DP1									
Sebum (μg/cm^2^)									
Model 1	141.2	(122.6, 159.8)	139.9	(123.0, 156.7)	149.3	(132.4, 166.2)	149.3	(130.9, 167.8)	0.459
Model 2	144.6	(125.0, 164.2)	139.1	(121.9, 156.3)	148.6	(131.4, 165.9)	147.4	(127.9, 166.9)	0.708
Skin hydration									
Model 1	63.8	(60.0, 67.6)	64.7	(61.2, 68.1)	63.4	(60.0, 66.9)	64.5	(60.7, 68.3)	0.935
Model 2	63.5	(59.6, 67.4)	64.0	(60.6, 67.4)	64.0	(60.6, 67.4)	64.9	(61.1, 68.8)	0.662
Skin pH									
Model 1	5.17	(4.96, 5.39)	5.18	(4.99, 5.38)	5.27	(5.07, 5.46)	5.31	(5.09, 5.52)	0.359
Model 2	5.11	(4.90, 5.33)	5.18	(4.99, 5.38)	5.24	(5.05, 5.44)	5.39	(5.17, 5.61)	0.106
DP2									
Sebum (μg/cm^2^)									
Model 1	154.0	(136.8, 171.3)	147.0	(129.9, 164.0)	130.5	(114.1, 146.9)	148.2	(129.6, 166.8)	0.379
Model 2	155.7	(138.1, 173.2)	145.3	(127.7, 162.9)	130.6	(113.7, 147.5)	148.2	(129.6, 166.9)	0.346
Skin hydration									
Model 1	66.9	(63.4, 70.3)	65.4	(62.0, 68.8)	64.2	(60.9, 67.5)	60.0	(56.2, 63.7)	0.017 *
Model 2	66.1	(62.7, 69.6)	66.7	(63.2, 70.2)	63.2	(59.8, 66.6)	60.4	(56.7, 64.1)	0.028 *
Skin pH									
Model 1	5.15	(4.95, 5.35)	5.33	(5.13, 5.53)	5.15	(4.96, 5.34)	5.31	(5.09, 5.52)	0.516
Model 2	5.17	(4.97, 5.37)	5.32	(5.12, 5.53)	5.16	(4.97, 5.35)	5.28	(5.06, 5.49)	0.708
DP3									
Sebum (μg/cm^2^)									
Model 1	153.8	(137.2, 170.5)	145.8	(129.2, 162.5)	141.0	(124.3, 157.6)	139.1	(122.5, 155.7)	0.190
Model 2	154.7	(138.0, 171.5)	148.0	(130.9, 165.0)	141.8	(125.1, 158.4)	135.3	(117.7, 152.8)	0.104
Skin hydration									
Model 1	64.5	(61.1, 67.9)	63.2	(59.8, 66.6)	64.5	(61.1, 68.0)	64.2	(60.8, 67.6)	0.940
Model 2	64.7	(61.4, 68.1)	62.4	(59.0, 65.8)	64.9	(61.6, 68.3)	64.4	(60.8, 67.9)	0.895
Skin pH									
Model 1	5.32	(5.14, 5.51)	5.33	(5.14, 5.51)	5.21	(5.02, 5.39)	5.08	(4.89, 5.26)	0.045 *
Model 2	5.29	(5.10, 5.48)	5.32	(5.13, 5.51)	5.18	(5.00, 5.37)	5.13	(4.93, 5.33)	0.186
DP4									
Sebum (μg/cm^2^)									
Model 1	129.8	(113.8, 145.8)	144.8	(128.9, 160.7)	141.2	(125.3, 157.1)	163.9	(148.0, 179.9)	0.008 **
Model 2	129.0	(112.0, 145.9)	146.8	(130.7, 163.0)	138.8	(122.7, 154.9)	165.2	(148.1, 182.2)	0.018 *
Skin hydration									
Model 1	65.7	(62.3, 69.0)	63.6	(60.3, 67.0)	61.9	(58.5, 65.2)	65.3	(61.9, 68.6)	0.713
Model 2	65.4	(61.8, 68.9)	63.7	(60.3, 67.0)	62.2	(58.9, 65.6)	65.2	(61.6, 68.7)	0.785
Skin pH									
Model 1	5.07	(4.88, 5.25)	5.22	(5.03, 5.40)	5.34	(5.15, 5.52)	5.31	(5.13, 5.50)	0.045 *
Model 2	5.08	(4.88, 5.27)	5.17	(4.98, 5.35)	5.35	(5.16, 5.54)	5.34	(5.14, 5.53)	0.039 *

DP, dietary pattern; * *p* < 0.05, ** *p* < 0.01; † Analysis of general linear model was performed using a linear trend test across quartiles of dietary pattern scores (Q1–Q4). Model 1 was adjusted only for energy intake. Model 2 was adjusted for Model 1 + sex, age, body mass index, smoking and physical activity.

**Table 4:** Skin health parameters across the quartiles of dietary pattern scores by sex †.

	Q1		Q2		Q3		Q4		*p* for Trend
	Mean	95% CI	Mean	95% CI	Mean	95% CI	Mean	95% CI	
Male (*n* = 44)									
DP1									
Sebum (μg/cm^2^)	158.7	(131.3, 186.0)	150.3	(122.5, 178.1)	138.1	(110.1, 166.1)	163.4	(133.6, 193.3)	0.946
Skin hydration	63.5	(58.1, 69.0)	63.9	(58.4, 69.5)	60.1	(58.4, 69.5)	62.8	(56.8, 68.8)	0.638
Skin pH	5.17	(4.94, 5.41)	5.31	(5.07, 5.55)	5.23	(4.99, 5.47)	5.45	(5.19, 5.70)	0.178
DP2									
Sebum (μg/cm^2^)	166.1	(139, 193.3)	165.4	(139.6, 191.1)	129.4	(103.6, 155.2)	149.6	(120.7, 178.5)	0.161
Skin hydration	68.3	(63.2, 73.5)	64.6	(59.8, 69.4)	60.4	(55.5, 65.2)	57.1	(51.7, 62.5)	0.003 **
Skin pH	5.14	(4.90, 5.39)	5.40	(5.16, 5.63)	5.31	(5.08, 5.55)	5.31	(5.05, 5.57)	0.469
DP3									
Sebum (μg/cm^2^)	157.8	(130.4, 185.3)	150.4	(122.7, 178.1)	149.7	(121.9, 177.5)	152.5	(124.1, 180.9)	0.771
Skin hydration	62.2	(56.8, 67.7)	62.5	(57.0, 67.9)	63.3	(57.8, 68.8)	62.5	(56.8, 68.1)	0.898
Skin pH	5.47	(5.25, 5.69)	5.21	(4.99, 5.43)	5.36	(5.14, 5.59)	5.12	(4.89, 5.35)	0.077
DP4									
Sebum (μg/cm^2^)	141.2	(115, 167.4)	160.3	(134.1, 186.6)	136.0	(110.1, 161.8)	173.0	(146.7, 199.2)	0.278
Skin hydration	63.9	(58.7, 69.2)	59.3	(54.1, 64.6)	61.1	(55.9, 66.2)	66.0	(60.8, 71.3)	0.504
Skin pH	5.03	(4.81, 5.25)	5.43	(5.21, 5.65)	5.34	(5.12, 5.55)	5.36	(5.14, 5.58)	0.090
Female (n = 40)									
DP1									
Sebum (μg/cm^2^)	132.0	(107.5, 156.4)	145.4	(118.7, 172.2)	130.2	(107.7, 152.7)	138.4	(108.4, 168.4)	0.814
Skin hydration	65.1	(60.7, 69.4)	67.7	(63.0, 72.5)	64.0	(60.0, 68.0)	66.3	(61.0, 71.7)	0.863
Skin pH	5.10	(4.73, 5.47)	5.18	(4.78, 5.59)	5.28	(4.94, 5.62)	5.12	(4.66, 5.57)	0.752
DP2									
Sebum (μg/cm^2^)	135.9	(111.2, 160.6)	137.9	(114.4, 161.5)	131.1	(108.1, 154.1)	141.0	(114.5, 167.6)	0.935
Skin hydration	65.2	(60.8, 69.6)	64.5	(60.3, 68.7)	65.8	(61.7, 69.9)	67.6	(62.8, 72.3)	0.460
Skin pH	5.14	(4.78, 5.51)	5.33	(4.98, 5.68)	5.04	(4.70, 5.38)	5.17	(4.78, 5.56)	0.772
DP3									
Sebum (μg/cm^2^)	149.9	(129.7, 170.0)	148.6	(128.2, 169.0)	137.1	(116.9, 157.3)	110.4	(90.0, 130.7)	0.009 **
Skin hydration	66.8	(62.9, 70.8)	63.5	(59.6, 67.5)	68.2	(64.3, 72.1)	64.5	(60.6, 68.5)	0.753
Skin pH	5.26	(4.92, 5.61)	5.18	(4.83, 5.53)	5.10	(4.76, 5.45)	5.13	(4.78, 5.48)	0.537
DP4									
Sebum (μg/cm^2^)	121.1	(100.5, 141.7)	136.7	(116.1, 157.4)	125.9	(106.7, 145.1)	162.2	(141.9, 182.4)	0.026 *
Skin hydration	67.1	(63.0, 71.2)	67.6	(63.5, 71.8)	64.8	(60.9, 68.6)	63.6	(59.6, 67.6)	0.143
Skin pH	5.22	(4.88, 5.55)	4.87	(4.53, 5.20)	5.25	(4.94, 5.56)	5.35	(5.02, 5.67)	0.309

DP, dietary pattern; * *p* < 0.05, ** *p* < 0.01; † Analysis of covariance was performed with a linear trend test across quartiles of dietary pattern scores (Q1–Q4) adjusting for energy intake, age, body mass index, smoking and physical activity.

**Table 5:** Correlation between nutrient intake and dietary pattern scores †.

Nutrient Intake	DP2		DP3		DP4	
	r	*p*-Value	r	*p*-Value	r	*p*-Value
Major nutrients						
Protein	−0.406	<0.001 ***	−0.078	0.483	0.237	0.031 *
Fat	−0.424	<0.001 ***	0.153	0.168	0.362	0.001 **
Carbohydrate	0.475	<0.001 ***	0.001	0.992	−0.426	<0.001 ***
Fiber	−0.220	0.046 *	0.556	<0.001 ***	−0.390	<0.001 ***
Vitamins						
Vitamin A	−0.454	<0.001 ***	0.396	<0.001 ***	0.132	0.234
Vitamin C	−0.225	0.041 *	0.685	<0.001 ***	0.218	0.047 *
Vitamin E	0.046	0.682	0.454	<0.001 ***	−0.007	0.946
Vitamin D	−0.300	0.006 **	0.627	<0.001 ***	0.281	0.010 *
Omega 6-fatty acids						
LA (C18:2n6)	−0.302	0.005 **	0.716	<0.001 ***	−0.254	0.020 *
GLA (C18:3n6)	−0.395	<0.001 ***	0.732	<0.001 ***	−0.224	0.042 *
DGLA (C20:3n6)	−0.165	0.137	0.692	<0.001 ***	−0.098	0.377
Amino acids						
Isoleucine	−0.221	0.044 *	−0.172	0.120	0.436	<0.001 ***
Leucine	−0.247	0.024 *	−0.225	0.040 *	0.470	<0.001 ***
Tryptophan	−0.120	0.279	0.124	0.263	0.415	<0.001 ***
Valine	−0.243	0.027 *	−0.206	0.061	0.456	<0.001 ***

DP, dietary pattern; LA, linoleic acid; GLA, gamma linolenic acid; DGLA, dihomo gamma linolenic acid; r, correlation coefficient. * *p* < 0.05, ** *p* < 0.01, *** *p* < 0.001. † Partial correlation analysis was applied to adjust for energy intake.
